# Children Are Likely to Suffer Most from Our Fossil Fuel Addiction

**DOI:** 10.1289/ehp.11173

**Published:** 2008-04-17

**Authors:** Frederica P. Perera

**Affiliations:** Columbia Center for Children’s Environmental Health, Mailman School of Public Health, Columbia University, New York, New York, USA

**Keywords:** asthma, cancer, climate change, energy, fetal and child development, fossil fuel, susceptibility

## Abstract

**Background:**

The periods of fetal and child development arguably represent the stages of greatest vulnerability to the dual impacts of fossil fuel combustion: the multiple toxic effects of emitted pollutants (polycyclic aromatic hydrocarbons, particles, sulfur oxides, nitrogen oxides, metals) and the broad health impacts of global climate change attributable in large part to carbon dioxide released by fossil fuel burning.

**Objectives:**

In this commentary I highlight current scientific evidence indicating that the fetus and young child are at heightened risk of developmental impairment, asthma, and cancer from fossil fuel pollutants and from the predicted effects of climate disruption such as heat waves, flooding, infectious disease, malnutrition, and trauma. Increased risk during early development derives from the inherently greater biologic vulnerability of the developing fetus and child and from their long future lifetime, during which early insults can potentially manifest as adult as well as childhood disease. I cite recent reports concluding that reducing dependence on fossil fuel and promoting clean and sustainable energy is economically feasible.

**Discussion:**

Although much has been written separately about the toxicity of fossil fuel burning emissions and the effects of climate change on health, these two faces of the problem have not been viewed together with a focus on the developing fetus and child. Adolescence and old age are also periods of vulnerability, but the potential for both immediate and long-term adverse effects is greatest when exposure occurs prenatally or in the early years.

**Conclusions:**

Consideration of the full spectrum of health risks to children from fossil fuel combustion underscores the urgent need for environmental and energy policies to reduce fossil fuel dependence and maximize the health benefits to this susceptible population. We do not have to leave our children a double legacy of ill health and ecologic disaster.

Although much has been written separately about the direct toxicity of fossil fuel burning emissions and the broad effects of climate change on health [see, e.g., the recent series in *Lancet* on energy and health (e.g., Wilkinson et al. 2007)], rarely are they viewed together with a focus on the very young as a susceptible population. In this commentary I highlight evidence that young children are likely to be at elevated risk of multiple immediate and long-term effects of emissions from fossil fuel combustion.

## Children at Risk from the Toxic and Carcinogenic Effects of Air Pollution from Fossil Fuel Combustion

As environmental health scientists, we have seen the direct damage inflicted on children in the United States and worldwide by our society’s addiction to fossil fuel. Fine particles, polycyclic aromatic hydrocarbons (PAHs), sulfur and nitrogen oxides, benzene and mercury emitted by coal-burning power plants, and diesel and gasoline-powered vehicles have been variously linked to infant mortality, lower birth weight, deficits in lung function, respiratory symptoms, childhood asthma, developmental disorders, and cancer (Bobak and Leon 1992; Gauderman et al. 2004; Grandjean and Landrigan 2006; Ha et al. 2003; Miller et al. 2004; Perera et al. 2006b; Šrám et al. 2005; Woodruff et al. 1997). The many observed adverse effects are not surprising, given the diversity of fossil fuel combustion products (Bernard et al. 2001); moreover, the same pollutant can exert multiple toxic effects. For example, *in utero* exposure to PAHs as a result of mothers breathing polluted air during pregnancy has been associated with lower birth weight, reduced birth head circumference, preterm birth, and small size for gestational age (Choi et al. 2006, 2008; Perera et al. 2003; Šrám et al. 2005). The same air pollutants have also been linked to developmental delay in U.S. and Chinese children (Perera et al. 2006b; Tang et al. 2006). Air pollution is not only an established trigger of asthma in children; but there is evidence that prenatal exposure to PAHs may be an early risk factor for the development of asthma (Miller et al. 2004). There is also a suggested link between PAHs and cancer (Bocskay et al. 2005).

These health effects represent a major societal and public health burden. A significant proportion of U.S. children 6–17 years of age are reported to have developmental problems including learning disabilities (11.5%), attention-deficit/hyperactivity disorder (8.8%), and behavioral problems (6.3%) (Blanchard et al. 2006). Asthma affects as many as 25% of children in certain inner-city communities in the United States (Nicholas et al. 2005), and the prevalence of asthma has increased throughout the developed world over the past 30 years (Beasley et al. 2003; National Institutes of Health 2001). Approximately 10,400 U.S. children under the age of 15 years were diagnosed with cancer in 2007 (American Cancer Society 2007). Although data are lacking on attributable risk of specific pollutants and relationships between trends in pollution and rates of disease, air pollutants such as lead and mercury are known to contribute to the burden of neurobehavioral disorders (Cheuk and Wong 2006; Lanphear et al. 2005; Stewart et al. 2006), and fine particles, ozone, diesel emissions, and PAHs are known or suspected contributors to childhood asthma (Etzel 2003; Strachan 2000).

Insults sustained early in development can have lifelong consequences. Some adult diseases can be launched *in utero* or in childhood. For example, exposure to air pollution in childhood may result in a reduction in lung function and ultimately to increased risk of chronic respiratory illness (Gauderman et al. 2000; Shea 2003) and greater susceptibility to cardiovascular disease in adulthood (Shea 2003). Similarly, several studies have indicated that genetic damage in the form of DNA adducts or chromosomal abnormalities can be acquired *in utero* as a result of air pollution exposure (Bocskay et al. 2005; Perera et al. 2005). Such types of genetic damage have been associated in prospective studies with increased risk of cancer and are considered biomarkers of increased cancer risk (Bonassi et al. 1995; Hagmar et al. 1994, 1998; Tang et al. 2002).

Epigenetic effects of developmental exposure to air pollutants have been less well studied. However, exposure to PAHs has been associated with epigenetic effects experimentally (Santangelo et al. 2002; Shin et al. 2005; Vercelli 2004; Wilson and Jones 1983; Wojciechowski and Meehan 1984), and prenatal exposure to PAHs in humans was shown to alter methylation status of a number of genes with known or suspected roles in asthma development (Perera et al. 2007). In addition, experimental studies, in some cases supported by human evidence, have demonstrated that epigenetic dysregulation resulting from *in utero* environmental exposures can lead to reproductive disorders and adult onset diseases such as cancer (Adam et al. 1985; Anway et al. 2005; Dolinoy et al. 2007; Feinberg and Tycko 2004; Ho et al. 2006).

A recent report from the American Lung Association noted that, although ozone levels have decreased in the United States since 2002, particle pollution has increased over that period, and coal-fired power plants are responsible for much of the increase in particle pollution in the eastern United States (American Lung Association 2007). The authors estimated that nearly half of the U.S. population (136 million) lives in counties that have unhealthful levels of either ozone (including 25 million children) or particle pollution (including 14 million children).

## Children at Risk from the Effects of Global Warming Due to Carbon Dioxide from Fossil Fuel Combustion

Children are also particularly vulnerable to the effects of global warming (Bunyavanich et al. 2003; Shea 2003). Anthropogenic carbon dioxide from fossil fuel burning is the most important climate-altering greenhouse gas (GHG) [Intergovernmental Panel on Climate Change (IPCC) 2007]. Fossil fuel use has been the primary source of CO_2_ concentrations since the preindustrial period (IPCC 2007). In the United States, energy-related activities account for three-quarters of human-generated GHG emissions, mostly in the form of CO_2_ emissions from burning fossil fuels [U.S. Environmental Protection Agency (EPA) 2008]. More than half the energy-related emissions come from large stationary sources such as power plants, while about a third (in the United States) comes from transportation (U.S. EPA 2008). In contrast, the livestock activities sector is responsible for approximately 18% of total anthropogenic GHG emissions measured in CO_2_ equivalent (Steinfeld et al. 2006). The average temperature of the earth is predicted to rise by 2–4°C (3.1–7.2°F) in this century (IPCC 2007). A temperature increase of this magnitude will bring more heat waves, flooding of coastal areas, famine, and forced migration (Bunyavanich et al. 2003; Haines and Patz 2004; IPCC 2001; Shea 2003). As a result of these changes, children are more at risk of heat stroke, drowning, malnutrition, diarrhea, allergies, infectious disease such as malaria and encephalitis, and psychological trauma (Bunyavanich et al. 2003; Haines and Patz 2004; IPCC 2001; Shea 2003).

Global warming also compounds the direct toxicity of fossil-fuel pollutants such as ozone, an important trigger of childhood asthma (Bernard et al. 2001). Ozone formation from volatile organic chemicals and nitrogen dioxide is accelerated at higher temperatures (Bernard et al. 2001). Another consequence of a warmer climate is increased plant growth and pollen production, and thus higher levels of natural allergens leading to more allergy and asthma in children (Bunyavanich et al. 2003).

## Heightened Vulnerability of the Fetus and Child: Poverty and Racism as Compounding Factors

The fetus and child are especially susceptible to air pollution and many other environmental contaminants because of their rapid development and immature defense systems; thus, they may be affected by levels of exposure that have no apparent effects in adults (Bearer 1995; Etzel and Balk 1999; Grandjean and Landrigan 2006; Perera et al. 2002, 2006a). For example, several studies have demonstrated the heightened susceptibility of the fetus to genetic damage in the form of carcinogen–DNA adducts (specifically PAH–DNA adducts) measured in white blood cells (Perera et al. 2004). Comparison of levels of adducts in paired maternal and umbilical cord white blood cells has found that, despite the estimated 10-fold lower PAH exposure of the fetus compared with the mother, the levels of PAH–DNA adducts were comparable (Perera et al. 2004). Moreover, although adolescence and old age are also periods of susceptibility to epigenetic reprogramming (Dolinoy et al. 2007), the epigenome is particularly susceptible to dysregulation from environmental factors during embryogenesis, when the elaborate DNA methylation patterning and chromatin structure required for normal tissue development are established (Dolinoy et al. 2007). Considering both their inherent biologic susceptibility and their long future lifetimes over which early insults can be manifest as chronic disease or cognitive impairment, the fetus and young child can be considered especially vulnerable and at risk of the multiple, cumulative, and long-term health effects of air pollution.

Poverty and racism compound the susceptibility of the fetus and child. Poor children, especially those in urban areas and developing countries, are most at risk, because the effects of toxic exposures are magnified by inadequate nutrition and psychosocial stress due to poverty or racism (Wood 2003). The shocking inequalities that now exist in children’s health within and between countries (Marmot 2006; Marmot et al. 1991; Waterston and Lenton 2000) will be exacerbated by global warming. The World Health Organization estimates that one-third of the global burden of disease is caused by environmental factors and that children < 5 years of age already bear > 40% of that burden, even though they represent only 10% of the world’s population (Prüss-Ustün and Corvalán 2006). That inequality will only get worse. Finally, perpetuation of fossil fuel burning violates the principle of intergenerational equity that no significant environmental burden should be inherited by future generations (World Commission on Environment and Development 1987).

Although more has been written about the heightened susceptibility of the fetus and child to toxic exposures, children also are likely to be especially susceptible to dehydration and heat stroke, malnutrition, diarrhea, allergies, malaria and encephalitis, and psychological trauma (Bunyavanich et al. 2003; Committee on Environmental Health 2007; Haines and Patz 2004; Shea 2003, 2007). The American Academy of Pediatrics Committee on Environmental Health noted: “Children represent a particularly vulnerable group that is likely to suffer disproportionately from both direct and indirect adverse health effects of climate change” (American Academy of Pediatrics 2007). For example, infants and young children are a high-risk group for heat-related deaths and hospitalizations, along with the elderly (Anonymous 2002). Children spend more time outdoors, particularly playing sports, which puts them at increased risk of heat stroke and heat exhaustion, as well as ultraviolet radiation–related basal cell carcinoma and malignant melanoma (American Academy of Pediatrics 2000). Because they lack specific immunity, children also experience disproportionately high levels of both morbidity and mortality from malaria; 75% of malaria deaths occur in children < 5 years of age. The young are also more susceptible to cerebral malaria, which can lead to lifelong neurologic damage in those who survive (Shea 2007). Once again, health and psychological damage occurring early in life can play out over the lifetime, manifesting as adult chronic disease or impairment.

## Solutions Exist

The most recent IPCC concluded that significant progress toward stabilizing or reducing global warming emissions can be achieved at relatively low cost using known technologies and practices currently available (IPCC 2007). A recent McKinsey report concluded that the United States could reduce GHG emissions in 2030 by 3.0–4.5 gigatons of CO_2_ equivalents using tested approaches and high-potential emerging technologies. The report stated: “Our research suggests that the net cost of achieving these levels of GHG abatement could be quite low on a societal basis” (McKinsey 2007).

These reports indicate that the cost of acting now to make power generation, transport, buildings, and appliances more efficient and to invest in alternative fuels and technologies would be minimal compared with the cost of doing nothing. The benefits of reducing air pollution and global warming would offset a substantial fraction of mitigation costs. These benefits include the individual and societal benefits of health and security extending multigenerationally and the monetary savings from fewer cases of children with asthma, developmental delay, cancer, heat stroke, drowning, malnutrition, diarrhea, allergies, and infectious disease.

## Conclusion

Summarizing the recent series of articles in *Lancet*, Richard Horton notes that “Policies to improve access to affordable clean energy should be pro-poor,” and that “Policies to reduce the progress and impact of climate change should explicitly aim to maximise health benefits and minimise health risks” (Horton 2007). Based on the present review, environmental and energy policies must also explicitly account for *all* the impacts of fossil fuel combustion on child health and development and maximize the health benefits to this susceptible population. Our addiction can be cured. We do not have to leave our children a double legacy of ill health and ecologic disaster.

## References

[b1-ehp0116-000987] Adam E, Kaufman RH, Adler-Storthz K, Melnick JL, Dreesman GR (1985). A prospective study of the association of herpes simplex virus and human papillomavirus infection with cervical neoplasia in women exposed to diethylstilbestrol *in utero*. Int J Cancer.

[b2-ehp0116-000987] American Academy of Pediatrics, Committee on Environmental Health (2007). Global Climate Change and Children’s Health. Pediatrics.

[b3-ehp0116-000987] American Academy of Pediatrics, Committee on Sports Medicine and Fitness (2000). Climatic heat stress and the exercising child and adolescent. Pediatrics.

[b4-ehp0116-000987] American Cancer Society (2007). Cancer Facts and Figures 2007.

[b5-ehp0116-000987] American Lung Association (2007). State of the Air 2007.

[b6-ehp0116-000987] Anonymous (2002). Heat-related deaths: four states, July–August 2001, and United States, 1979–1999. MMWR Morb Mortal Wkly Rep.

[b7-ehp0116-000987] Anway MD, Cupp AS, Uzumcu M, Skinner MK (2005). Epigenetic transgenerational actions of endocrine disruptors and male fertility. Science.

[b8-ehp0116-000987] Bearer CF (1995). Environmental health hazards: how children are different from adults. Future Child.

[b9-ehp0116-000987] Beasley R, Ellwood P, Asher I (2003). International patterns of the prevalence of pediatric asthma: the ISAAC program. Peadiatr Clin North Am.

[b10-ehp0116-000987] Bernard SM, Samet JM, Grambsch A, Ebi KL, Romieu I (2001). The potential impacts of climate variability and change on air pollution-related health effects in the United States. Environ Health Perspect.

[b11-ehp0116-000987] Blanchard L, Gurka M, Blackman J (2006). Emotional, developmental, and behavioral health of American children and their families: a report from the 2003 National Survey of Children’s Health Emotional, Developmental, and Behavioral Health of American Children. Pediatrics.

[b12-ehp0116-000987] Bobak M, Leon DA (1992). Air pollution and infant mortality in the Czech Republic, 1986–88. Lancet.

[b13-ehp0116-000987] Bocskay KA, Tang D, Orjuela MA, Xinhua L, Warburton DP, Perera FP (2005). Chromosomal aberrations in cord blood are associated with prenatal exposure to carcinogenic polycyclic aromatic hydrocarbons. Cancer Epidemiol Biomarker Prev.

[b14-ehp0116-000987] Bonassi S, Abbondandolo A, Camurri L, Dal Pra L, De Ferrari M, Degrassi F (1995). Are chromosome aberrations in circulating lymphocytes predictive of future cancer onset in humans? Preliminary results of an Italian cohort study. Cancer Genet Cytogenet.

[b15-ehp0116-000987] Bunyavanich S, Landrigan CP, McMichael AJ, Epstein PR (2003). The impact of climate change on child health. Ambul Pediatr.

[b16-ehp0116-000987] Cheuk D, Wong V (2006). Attention-deficit hyperactivity disorder and blood mercury level: a case-control study in Chinese children. Neuropediatrics.

[b17-ehp0116-000987] Choi H, Jedrychowski W, Spengler J, Camann DE, Whyatt RM, Rauh V (2006). International studies of prenatal exposure to polycyclic aromatic hydrocarbons and fetal growth. Environ Health Perspect.

[b18-ehp0116-000987] Choi H, Rauh V, Garfinkel R, Tu Y, Perera FP (2008). Prenatal exposure to airborne polycyclic aromatic hydrocarbons and risk of intrauterine growth restriction. Environ Health Perspect.

[b19-ehp0116-000987] Dolinoy DC, Weidman JR, Jirtle RL (2007). Epigenetic gene regulation: linking early developmental environment to adult disease. Reprod Toxicol.

[b20-ehp0116-000987] Etzel RA (2003). How environmental exposures influence the development and exacerbation of asthma. Pediatrics.

[b21-ehp0116-000987] Etzel RA, Balk SJ (1999). Handbook of Pediatric Environmental Health.

[b22-ehp0116-000987] Feinberg AP, Tycko B (2004). The history of cancer epigenetics. Nat Rev Cancer.

[b23-ehp0116-000987] Gauderman WJ, Avol E, Gilliland F, Vora H, Thomas D, Berhane K (2004). The effect of air pollution on lung development from 10 to 18 years of age. N Engl J Med.

[b24-ehp0116-000987] Gauderman WJ, McConnell R, Gilliland F, London S, Thomas D, Avol E (2000). Association between air pollution and lung function growth in southern California children. Am J Respir Crit Care Med.

[b25-ehp0116-000987] Grandjean P, Landrigan PJ (2006). Developmental neurotoxicity of industrial chemicals. Lancet.

[b26-ehp0116-000987] Ha EH, Lee JT, Kim H, Hong YC, Lee BE, Park HS (2003). Infant susceptibility of mortality to air pollution in Seoul, South Korea. Pediatrics.

[b27-ehp0116-000987] Hagmar L, Bonassi S, Stromberg U, Brogger A, Knudsen LE, Norppa H (1998). Chromosomal aberrations in lymphocytes predict human cancer: a report from the European Study Group on Cytogenetic Biomarkers and Health (EDCH). Cancer Res.

[b28-ehp0116-000987] Hagmar L, Brogger A, Hansteen IL, Heim S, Hogstedt B, Knudsen L (1994). Cancer risk in humans predicted by increased levels of chromosomal aberrations in lymphocytes: Nordic Study group on the health risk of chromosome damage. Cancer Res.

[b29-ehp0116-000987] Haines A, Patz JA (2004). Health effects of climate change. JAMA.

[b30-ehp0116-000987] Ho SM, Tang WY, Belmonte de Frausto J, Prins GS (2006). Developmental exposure to estradiol and bisphenol A increases susceptibility to prostate carcinogenesis and epi-genetically regulates phosphodiesterase type 4 variant 4. Cancer Res.

[b31-ehp0116-000987] Horton R (2007). Righting the balance: energy for health. Lancet.

[b32-ehp0116-000987] IPCC (2001). IPCC Third Assessment Report.

[b33-ehp0116-000987] IPCC (2007). Fourth Assessment Report.

[b34-ehp0116-000987] Lanphear BP, Hornung R, Khoury J, Yolton K, Baghurst P, Bellinger DC (2005). Low-level environmental lead exposure and children’s intellectual function: an international pooled analysis. Environ Health Perspect.

[b35-ehp0116-000987] Marmot M (2006). Harveian Oration: Health in an unequal world. Lancet.

[b36-ehp0116-000987] Marmot MG, Smith GD, Stansfeld S, Patel C, North F, Head J (1991). Health inequalities among British civil servants: The Whitehall II Study. Lancet.

[b37-ehp0116-000987] McKinsey CA (2007). Reducing U.S. Greenhouse Gas Emissions: How much at what cost? Greenhouse Gas Report.

[b38-ehp0116-000987] Miller RL, Garfinkel R, Horton M, Camann D, Perera FP, Whyatt RM (2004). Polycyclic aromatic hydrocarbons, environmental tobacco smoke, and respiratory symptoms in an inner-city birth cohort. Chest.

[b39-ehp0116-000987] National Institutes of Health (2001). NHLBI Reports New Asthma Data for World Asthma Day 2001: Asthma Still a Problem but More Groups Fighting It [Press Release].

[b40-ehp0116-000987] Nicholas SW, Jean-Louis B, Ortiz B, Northridge M, Shoemaker K, Vaughan R (2005). Addressing the childhood asthma crisis in Harlem: the Harlem Children’s Zone Asthma Initiative. Am J Public Health.

[b41-ehp0116-000987] Perera F, Tang D, Whyatt R, Lederman SA, Jedrychowski W (2005). DNA damage from polycyclic aromatic hydrocarbons measured by benzo[*a*]pyrene-DNA adducts in mothers and newborns from northern Manhattan, the World Trade Center area, Poland, and China. Cancer Epidemiol Biomarkers Prev.

[b42-ehp0116-000987] Perera F, Viswanathan S, Whyatt R, Tang D, Miller RL, Rauh V (2006a). Children’s environmental health research—highlights from the Columbia Center for Children’s Environmental Health. Ann NY Acad Sci.

[b43-ehp0116-000987] Perera FP, Illman SM, Kinney PL, Whyatt RM, Kelvin EA, Shepard P (2002). The challenge of preventing environmentally related disease in young children: community-based research in New York City. Environ Health Perspect.

[b44-ehp0116-000987] Perera FP, Rauh V, Tsai WY, Kinney P, Camann D, Barr D (2003). Effects of transplacental exposure to environmental pollutants on birth outcomes in a multi-ethnic population. Environ Health Perspect.

[b45-ehp0116-000987] Perera FP, Rauh V, Whyatt RM, Tsai WY, Tang D, Diaz D (2006b). Effect of prenatal exposure to airborne polycyclic aromatic hydrocarbons on neurodevelopment in the first 3 years of life among inner-city children. Environ Health Perspect.

[b46-ehp0116-000987] Perera FP, Tang D, Whyatt RM, Lederman SA, Jedrychowski W (2004). Comparison of PAH-DNA adducts in four populations of mothers and newborns in the U.S., Poland and China.

[b47-ehp0116-000987] Perera FP, Tang W, Herbstman J, Edwards SC, Tang D, Ho S-M (2007). Prenatal exposure to airborne polycyclic aromatic hydrocarbons and alterations in DNA methylation in cord blood.

[b48-ehp0116-000987] Prüss-Ustün A, Corvalán C (2006). Preventing Disease through Healthy Environments: Towards an Estimate of the Environmental Burden of Disease.

[b49-ehp0116-000987] Santangelo S, Cousins DJ, Winkelmann NE, Staynov DZ (2002). DNA methylation changes at human Th2 cytokine genes coincide with DNase I hypersensitive site formation during CD4(+) T cell differentiation. J Immunol.

[b50-ehp0116-000987] Shea KM (2003). Global environmental change and children’s health: understanding the challenges and finding solutions. J Pediatr.

[b51-ehp0116-000987] Shea KM (2007). Global climate change and children’s health. Pediatrics.

[b52-ehp0116-000987] Shin HJ, Park HY, Jeong SJ, Park HW, Kim YK, Cho SH (2005). STAT4 expression in human T cells is regulated by DNA methylation but not by promoter polymorphism. J Immunol.

[b53-ehp0116-000987] Šrám RJ, Binková B, Dejmek J, Bobak M (2005). Ambient air pollution and pregnancy outcomes: a review of the literature. Environ Health Perspect.

[b54-ehp0116-000987] Steinfeld H, Gerber P, Wassenaar T, Castel V, Rosales M, de Haan C (2006). Livestock’s Long Shadow: Environmental Issues and Options.

[b55-ehp0116-000987] Stewart PW, Sargent DM, Reihman J, Gump BB, Lonky E, Darvill T (2006). Response inhibition during differential reinforcement of low rates (DRL) schedules may be sensitive to low-level polychlorinated biphenyl, methylmercury, and lead exposure in children. Environ Health Perspect.

[b56-ehp0116-000987] Strachan DP (2000). The role of environmental factors in asthma. Br Med Bull.

[b57-ehp0116-000987] Tang D, Cho S, Rundle A, Chen S, Phillips D, Zhou J (2002). Polymorphisms in the DNA repair enzyme XPD are associated with increased levels of PAH-DNA adducts in a case-control study of breast cancer. Breast Cancer Res Treat.

[b58-ehp0116-000987] Tang D, Li TY, Liu JJ, Chen YH, Qu L, Perera FP (2006). PAH-DNA Adducts in cord blood and fetal and child development in a Chinese cohort. Environ Health Perspect.

[b59-ehp0116-000987] U.S. EPA (2008). Basic Information.

[b60-ehp0116-000987] Vercelli D (2004). Genetics, epigenetics, and the environment: switching, buffering, releasing. J Allergy Clin Immunol.

[b61-ehp0116-000987] Waterston T, Lenton S (2000). Public health: sustainable development, human induced global climate change, and the health of children. Arch Dis Child.

[b62-ehp0116-000987] Wilkinson P, Smith K, Beevers S, Tonne C, Oreszczyn T (2007). Energy, energy efficiency, and the built environment. Lancet.

[b63-ehp0116-000987] Wilson VL, Jones PA (1983). Inhibition of DNA methylation by chemical carcinogens *in vitro*. Cell.

[b64-ehp0116-000987] Wojciechowski M, Meehan T (1984). Inhibition of DNA methyl-transferases *in vitro* by benzo[*a*]pyrene diol epoxide-modified substrates. J Biol Chem.

[b65-ehp0116-000987] Wood D (2003). Effect of child and family poverty on child health in the United States. Pediatrics.

[b66-ehp0116-000987] Woodruff TJ, Grillo J, Schoendorf KC (1997). The relationship between selected causes of postneonatal infant mortality and particulate air pollution in the United States. Environ Health Perspect.

[b67-ehp0116-000987] World Commission on Environment and Development (1987). Our Common Future (Brundtland Report).

